# ATTIRE: Albumin To prevenT Infection in chronic liveR failurE: study protocol for a single-arm feasibility trial

**DOI:** 10.1136/bmjopen-2015-010132

**Published:** 2016-01-25

**Authors:** Louise China, Nicola Muirhead, Simon S Skene, Zainib Shabir, Roel PH De Maeyer, Alexander AN Maini, Derek W Gilroy, Alastair J O'Brien

**Affiliations:** 1 Division of Medicine, University College London (UCL), London, UK; 2 Comprehensive Clinical Trials Unit, University College London (UCL), London, UK

**Keywords:** Albumin, Liver Cirrhosis, Prostaglandin E2

## Abstract

**Introduction:**

Circulating prostaglandin E_2_ levels are elevated in acutely decompensated cirrhosis and have been shown to contribute to immune suppression. Albumin binds and inactivates this hormone. Human albumin solution could thus be repurposed as an immune restorative drug in these patients.

This feasibility study aims to determine whether it is possible and safe to restore serum albumin to >30 g/L and maintain it at this level in patients admitted with acute decompensated cirrhosis using repeated 20% human albumin infusions according to daily serum albumin levels.

**Methods and analysis:**

Albumin To prevenT Infection in chronic liveR failurE (ATTIRE) stage 1 is a multicentre, open label dose feasibility trial. Patients with acutely decompensated cirrhosis admitted to hospital with a serum albumin of <30 g/L are eligible, subject to exclusion criteria. Daily intravenous human albumin solution will be infused, according to serum albumin levels, for up to 14 days or discharge in all patients. The primary end point is daily serum albumin levels for the duration of the treatment period and the secondary end point is plasma-induced macrophage dysfunction. The trial will recruit 80 patients. Outcomes will be used to assist with study design for an 866 patient randomised controlled trial at more than 30 sites across the UK.

**Ethics and dissemination:**

Research ethics approval was given by the London-Brent research ethics committee (ref: 15/LO/0104). The clinical trials authorisation was issued by the medicines and healthcare products regulatory agency (ref: 20363/0350/001-0001).

**Results:**

Will be disseminated through peer reviewed journals and international conferences. Recruitment of the first participant occurred on 26/05/2015.

**Trial registration number:**

The trial is registered with the European Medicines Agency (EudraCT 2014-002300-24) and has been adopted by the NIHR (ISRCTN 14174793). This manuscript refers to V.4.0 of the protocol; Pre-results.

**Strengths and limitations of this study**

- This study will demonstrate feasibility and safety of targeted albumin dosing in patients with acutely decompensated liver cirrhosis prior to a large randomised controlled trial (RCT).
- Biomarker end point provides validation of a novel biological assay for immune dysfunction.
- Outcomes will ensure optimal study protocol design for the RCT.
- Feasibility study therefore not randomised or powered to detect clinically relevant beneficial outcomes.

## Introduction

Liver disease is the only major cause of mortality currently increasing in the UK and is the fifth most common cause of death.^1^ These deaths are predicted to double over the next 20 years.^2^

Patients with symptoms of liver failure secondary to cirrhosis are described as acute decompensation (AD) patients. They are highly prone to bacterial infection^3^ secondary to immune dysfunction,^4^ with nosocomial (hospital-acquired) infection rates of 35% compared to 5% in non-cirrhotic patients.^5^ ^6^ Of those that develop infection with organ dysfunction, 60–95% die, often following prolonged intensive care unit (ICU) admission.^7^ There is, however, no medical strategy to restore immune competence.

It has been demonstrated that elevated circulating prostaglandin E_2_ (PGE_2_) levels contribute to immune suppression in AD patients.^8^ The plasma protein albumin binds and catalyses inactivation of PGE_2._^9^ Albumin is synthesised in the liver and levels fall as the synthetic function of the liver declines in advanced cirrhosis, making PGE_2_ more bioavailable. In addition the binding capacity of endogenous albumin is known to be defective in cirrhosis.^10^ ^11^ We found a serum albumin of <30 g/L predicted plasma-induced macrophage dysfunction in a small cohort of AD patients^8^ and this was reversed when albumin levels were increased to >30 g/L.

We propose a novel strategy to repurpose 20% human albumin solution (HAS) as an immune restorative drug in AD patients with the aim of maintaining serum albumin at near normal levels.

Albumin To prevenT Infection in chronic liveR failurE (ATTIRE) incorporates a phase II single-arm multicentre feasibility trial (n=80) prior to a phase III randomised controlled trial (RCT) (n=866) assessing the impact of treatment on the incidence of nosocomial infections, organ dysfunction and mortality in patients admitted to hospital with AD of liver cirrhosis.

This feasibility trial aims to verify that daily intravenous human albumin infusions will restore serum albumin levels to near normal in AD patients, that this is safe and that there is physician equipoise prior to proceeding to a large RCT. Despite multiple studies, including systematic reviews,^12^ ^13^ evaluating albumin in septic intensive care patients there is a lack of interventional RCTs in patients with liver cirrhosis in which the mechanism of albumin's action is different.^14–16^ To date there has not been an albumin dosing trial aimed at increasing serum albumin levels in this context therefore it was essential that this was completed before proceeding to a large, interventional RCT. We shall also examine the effects on patient plasma-induced macrophage dysfunction using assays developed within our laboratory^8^ which will validate the proposed mechanism of albumin's action in patients with chronic liver failure.

## Methods and analysis

### Primary objective

**Figure 1:**
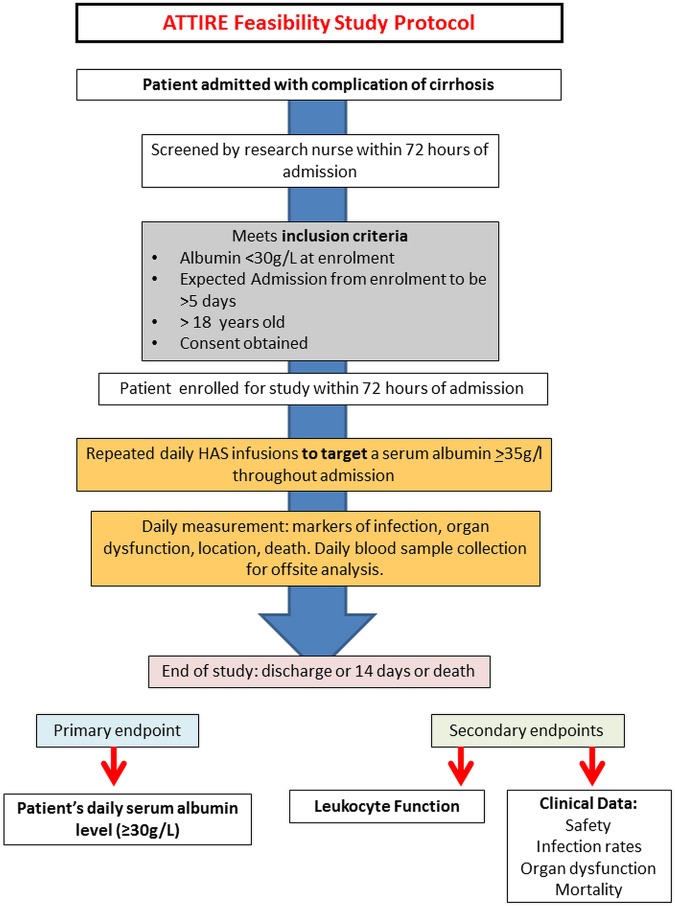
Protocol for ATTIRE phase II feasibility trial. ATTIRE, Albumin To prevenT Infection in chronic liveR failurE.

To determine whether it is possible to restore to and maintain serum albumin at >30 g/L in patients admitted with AD using repeated 20% HAS infusions according to daily measured serum albumin levels (figure 1).

### Secondary objective

We shall assess patient plasma-induced macrophage dysfunction (as an indicator of immune suppression) in AD patients on the day of recruitment and during HAS treatment to determine whether this is substantially improved following albumin infusion.^17^

### Trial design

This is a multicentre, open label single-arm feasibility trial in which all patients will be treated with 20% HAS to target levels above 30 g/L. Sequential patients admitted to 10 UK participating hospitals with a clinical diagnosis of cirrhosis and AD will be screened using the inclusion and exclusion criteria (table 1).

**Table 1:** Patient selection criteria

Patient inclusion criteria	Patient exclusion criteria
All patients admitted to hospital with acute onset or worsening of complications of cirrhosis	Advanced hepatocellular carcinoma with life expectancy of less than 8 weeks
Over 18 years of age	Patients who will receive palliative treatment only during their hospital admission
Predicted hospital admission >5 days at trial enrolment, which must be within 72 h of admission	Patients who are pregnant
Serum albumin <30 g/L at screening	Known or suspected severe cardiac dysfunction
Documented informed consent to participate (or consent given by a legal representative)	Any clinical condition which the investigator considers would make the patient unsuitable for the trial
	The patient has been involved in a clinical trial of Investigational Medicinal Products (IMPs) within the previous 30 days that would impact on their participation in this study
	Trial investigators unable to identify the patient (by NHS number)

NHS, National Health Service.

### Clinical trial end points

The primary end point is daily serum albumin level for the duration of the treatment period (maximum of 14 days or when the patient is considered fit for discharge if less than 14 days).

The key secondary end point is patient plasma-induced macrophage dysfunction assessed by our laboratory-based assays.^8^ Immune function is an extremely complex process for which there is no simple test or assay. During inflammation, monocytes move quickly to sites of tissue infection and differentiate into macrophages to elicit an immune response. Numerous studies have demonstrated the role of monocyte deactivation in cirrhosis associated immune suppression.^18–20^ It is, however, impractical to perform blinded, standardised, biological assays using fresh monocytes from 10 sites spread throughout the UK. As it has been demonstrated that circulating plasma mediators are responsible for monocyte and neutrophil dysfunction,^21^ ^22^ we developed an assay in which stored plasma from AD patients is added to macrophages from healthy donors.^8^ This permits testing of patient samples from multiple sites at the same time in a controlled fashion.

We have selected macrophage production of the proinflammatory cytokine tumour necrosis factor α (TNF-α) as our immune-readout as this has been validated as a biomarker of monocyte function in critical illness. Reduced capacity to produce TNF-α is associated with adverse outcomes following sepsis.^23^ ^24^

We will stimulate human monocyte-derived macrophages with lipopolysaccharide (LPS) in the presence of 25% patient plasma pretreatment and post-treatment, measuring TNF-α production. LPS simulates a bacterial infectious stimulus. Improvement in macrophage function will be defined as an increase in LPS-induced TNF-α production. Plasma from healthy controls will also be used as a comparator.

Other secondary end points include rates of infection, organ dysfunction and mortality during the 14-day treatment period. Clinical data will also be collected regarding safety, type of infection, antibiotic prescribing, total amount of fluid administered, ICU admission and duration of hospital stay.

### Patient population

This will include all patients admitted to hospital with complications of liver cirrhosis and serum albumin <30 g/L, aged over 18 years with anticipated hospital length of stay of five or more days at trial enrolment, which should be no later than 72 h from admission. This is subject to exclusion criteria as detailed in table 1. The diagnosis of cirrhosis will be performed by the clinical team as per standard UK practice and does not require liver biopsy or imaging.

### Consent

Patient information sheets (see online supplementary appendix 1) will be given to and discussed with potential patients before consent is sought. Informed consent will be obtained from each participant or their legal representative. Patients who lack mental capacity, for any reason, are not excluded from the trial. An important subgroup of patients will have hepatic encephalopathy and these patients may lack capacity to consent. However, these patients may be among those that receive maximum benefit from the intervention.^25–27^ In this case consent will be sought from an appropriate legal representative independent of the research team as per current UK clinical trials regulations.^28^

### Intervention

All patients will receive a daily infusion of 20% HAS intravenously (100mL/h) for a maximum of 14 days or until discharge (if less than 14 days). The volume of HAS prescribed each day will be determined by the patient's serum albumin level on that day.

Table 2 shows a suggested dosing protocol for albumin administration. This is based on the reported regimen used in the ALBIOS study^29^ and clinical experience as there are no prior albumin dose–increment studies in cirrhosis patients. In ALBIOS^29^ patients with a very low albumin (<20 g/L) incremented to a higher value within 4–5 days therefore we would expect 20% HAS requirements, as according to our trial protocol, to decrease after a few days with a subsequent decrease in cost and time of administration. However, there may be a subgroup of non-responders. We expect these patients to be very unwell and careful monitoring of side effects with on-going albumin infusion may warrant cessation of the trial intervention in this group. If this is the case additional time-to-increment advice may be added to the RCT protocol.

**Table 2:** Dosing protocol for 20% HAS administration (amounts per day) as advised by measured serum albumin level on that day

Patient's serum albumin level (g/L)	Amount of 20% HAS to be administered (mL)
≥35	None
30–34	100
26–29	200
20–25	300
<20	400

HAS, human albumin solution.

Differing regimens may be used to cover large volume paracentesis (8 g of albumin/L of ascites drained) or treat hepatorenal syndrome (1 g of albumin/kg of body weight) as per international guidelines^30^ ^31^ but HAS *must* be prescribed and given if serum albumin<35 g/L**.** All variations will be recorded in the patient's daily Case Report Form (CRF).

### Evaluations during and after treatment

Clinical, biochemical and microbiological data will be collected daily during the trial treatment period (see online supplementary appendix 2) using information from hospital notes that is recorded as standard of care. There is no follow-up beyond the treatment period. The blood samples collected for immune function tests will be analysed in a blinded fashion at a central site.

### Statistical considerations

#### Sample size

The primary purpose of this Feasibility Trial is to demonstrate that repeated 20% HAS infusions can raise and maintain serum albumin at ≥30 g/L in liver cirrhosis patients presenting with AD.

Eighty patients will be recruited. Success would be demonstrated if 60% of patients were able to achieve and maintain serum albumin levels at or above 30 g/L on at least 1/3 of days in which the level is recorded. We believe that there will be a subgroup of patients with very low albumin levels who may not be able to achieve this end point which has influenced our definition of success. If our assumptions are correct these patients will be excluded from RCT recruitment. With 72 evaluable patients (allowing for 10% loss-to-follow-up/withdrawal) the probability of achieving 44 or more ‘successes’ is around 80% assuming that each patient has a 65% chance of attaining the required level.

The trial will be performed at 10 sites. 8–10 patients per site will allow identification of any variability in the delivery of the albumin-targeting dose protocol between centres. It will be compulsory to record reason for protocol variation in the daily CRF.

#### Statistical evaluation

As this is a feasibility trial the emphasis will be on producing relevant data summaries rather than on formal modelling or hypothesis testing. This will support the IDMC and TSC in deciding whether to recommend proceeding with the RCT.

##### Primary outcome

Serum albumin levels will be summarised for each of days 1–15 by table, mean±SD and graph (median level vs day with superimposed bars displaying IQR). The numbers of patients observed on each day will be noted. Day 1 will represent the baseline serum albumin level before the first administration of 20% HAS according to the protocol.

The number of patients on each day whose serum albumin level exceeds 30 g/L will be noted as a percentage of those evaluated, together with the overall percentage of patients whose serum albumin level exceeds 30 g/L on at least 1/3 of the days on which it is recorded.

##### Secondary outcomes

Albumin's ex vivo impact on immune function will be determined by comparing macrophage function in the presence of patient plasma using our laboratory assays when their albumin level is <30 g/L compared to ≥30 g/L. A substantial improvement in macrophage function is expected following treatment with albumin.

Information shall be summarised regarding the total volume of albumin infused and the total amount of fluid administered, days in ICU and duration of hospital stay, together with the rates of nosocomial infections, organ dysfunction and in-hospital mortality which are the component elements of the composite end point for the RCT. Safety will be assessed by consideration of the number of SAEs reported during the trial.

Data will be further summarised within ‘groups’ defined by baseline serum albumin levels (<20, 20–25 and 26–29 g/L) to investigate whether there are any apparent differences in outcome by group. This subgroup analysis is exploratory, since we are not powered to detect differences, but may be useful in identifying whether any amendments are necessary to the protocol for the RCT.

## Discussion

ATTIRE is a UK multicentre trial that aims to evaluate the repurposing of HAS as an immune restorative drug. This protocol describes the feasibility trial which will determine if it is possible and safe to raise and maintain AD patients’ serum albumin levels to >30 g/L.

In liver cirrhosis current evidence-based guidance^31–33^ advocates the use of HAS in large volume paracentesis,^34^ hepatorenal syndrome^35^ and spontaneous bacterial peritonitis.^36^ ^37^ To date there has not been an albumin dosing trial aimed at increasing serum albumin levels. Therefore it was essential that this study was completed before proceeding to a large, interventional RCT.

Studies evaluating the safety of HAS infusions have generally shown it to be a safe treatment.^12^ ^13^ ^29^ ^34^ The main concerns in the cirrhotic population are related to volume overload leading to pulmonary oedema and increase in portal pressure leading to variceal bleeding. A recent interventional trial in septic AD patients reported an 8.3% rate of pulmonary oedema in the albumin treatment group.^38^ However, the weight based albumin dosing regimen in this study led to much larger daily volumes of albumin being prescribed than suggested in our protocol. This and other studies in cirrhosis have not reported an increased incidence in variceal bleeding.^34^ ^37^ ^39^

We shall also correlate our primary end point of a numerical increase in albumin levels with a biological outcome measuring markers of immune function ex vivo. This aims to verify that increasing serum albumin above 30 g/L is associated with an improvement in patient plasma-induced macrophage dysfunction ex vivo in a much larger patient cohort than already shown.^8^ These outcomes will be used to move forward and if appropriate modify the protocol for the ATTIRE RCT.

### Ethics and dissemination

This research group will involve a potentially vulnerable patient group that have hepatic encephalopathy and therefore lack the capacity to consent. However, patients with encephalopathy are at high risk of infection and could be those that potentially receive maximal benefit from the intervention and therefore should not be denied access to the trial treatment. We have undertaken steps to ensure these patients are appropriately recruited to the trial (described in ‘Consent’ section) and provided individual site training.

Research Ethics positive opinion was given by the London-Brent Research Ethics Committee (ref: 15/LO/0104) which specialise in trials involving patients who lack the capacity to consent.

The Clinical Trials Authorisation was issued by the Medicines and Healthcare products Regulatory Agency (MHRA, ref: 20363/0350/001-0001). The trial is registered with the European Medicines Agency (EudraCT 2014-002300-24) and has been adopted by the NIHR. Recruitment started in May 2015 and will finish by November 2015. Assuming feasibility milestones have been met a 866 patient phase III randomised control trial (ATTIRE stage 2) will start at the end of 2015 randomising patients to daily HAS infusion or routine standard of care.

Study findings will be disseminated through peer reviewed publications and international conference presentations. They will also be used to generate the study protocol for a large interventional RCT (ATTIRE stage 2).

### Trial funding and sponsor

The work is supported by the Health Innovation Challenge fund (Wellcome Trust and Department of Health) award number 164699. The trial sponsor is UCL with trial management activities conducted by the UCL Comprehensive Clinical Trials Unit.

## Trial management and monitoring

### Research Steering Group

The Research Steering Group (RSG) operates on behalf of the funders to ensure that appropriate milestones have been met in the delivery of the trial. It consists the CI, an independent expert and representatives of the Welcome Trust and Department of Health.

### Trial Management Group

The Trial Management Group (TMG) comprises the CI, Clinical Research Fellow, Clinical Project Manager, Trial Statistician, Trial Manager, Data Manager, Health Economist and five trial site PIs. The TMG is responsible for developing the design, co-ordination and strategic management of the trial.

### Trial Steering Committee

The Trial Steering Committee (TSC) is the independent group responsible for oversight of the trial in order to safeguard the interests of trial patients. The TSC provides advice to the CI, Clinical Trials Unit (CTU), funder and sponsor on all aspects of the trial through its independent chair.

### Independent Data Monitoring Committee

The Independent Data Monitoring Committee (IDMC) is responsible for safeguarding the interests of trial patients, monitoring the accumulating data and making recommendations to the TSC on whether the trial should continue as planned. It comprises a clinical chair (independent hepatologist), independent gastroenterologist and an independent statistician all with expertise in Clinical Trials.
